# Roles, Actions, and Therapeutic Potential of Specialized Pro-resolving Lipid Mediators for the Treatment of Inflammation in Cystic Fibrosis

**DOI:** 10.3389/fphar.2019.00252

**Published:** 2019-04-02

**Authors:** Antonio Recchiuti, Domenico Mattoscio, Elisa Isopi

**Affiliations:** ^1^ Department of Medical, Oral and Biotechnological Science, Università “G. d’Annunzio” Chieti-Pescara, Chieti, Italy; ^2^ Centro di Scienze dell’Invecchiamento e Medicina Traslazionale (CeSI-MeT), Università “G. d’Annunzio” Chieti-Pescara, Chieti, Italy

**Keywords:** resolution, lipid mediator, pharmacology, macrophages, lung inflammation and fibrosis, homeostasis, chronic infections, *Pseudomonas aeruginosa*

## Abstract

Non-resolving inflammation is the main mechanism of morbidity and mortality among patients suffering from cystic fibrosis (CF), the most common life-threatening human genetic disease. Resolution of inflammation is an active process timely controlled by endogenous specialized pro-resolving lipid mediators (SPMs) produced locally in inflammatory loci to restrain this innate response, prevent further damages to the host, and permit return to homeostasis. Lipoxins, resolvins, protectins, and maresins are SPM derived from polyunsaturated fatty acids that limit excessive leukocyte infiltration and pro-inflammatory signals, stimulate innate microbial killing, and enhance resolution. Their unique chemical structures, receptors, and bioactions are being elucidated. Accruing data indicate that SPMs carry protective functions against unrelenting inflammation and infections in preclinical models and human CF systems. Here, we reviewed their roles and actions in controlling resolution of inflammation, evidence for their impairment in CF, and proofs of principle for their exploitation as innovative, non-immunosuppressive drugs to address inflammation and infections in CF.

## Acute Inflammation and Resolution: Definitions and Key Mechanisms

**Figure 1:**
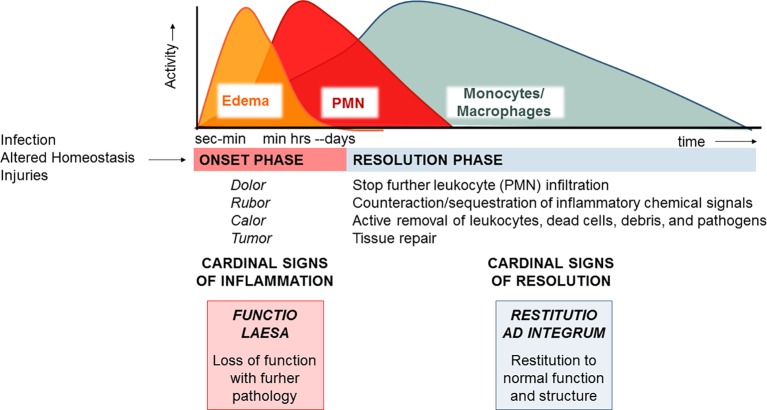
The acute inflammatory response and its ideal outcome: essential steps, mechanisms, and definitions. Injury, infections, or dysregulated homeostasis ignites the acute inflammatory response that is normally a host protective mechanism. The first event in acute inflammation is edema formation, followed by infiltration of PMN, and then monocyte and macrophages that clear PMN leading to resolution, which is essential for ensuring host protection and sparing from tissue damage.

Acute inflammation is a protective process arising in vascularized tissues upon damages, altered homeostasis, and infections. Its macroscopic hallmarks, or “cardinal signs,” identified by Celsus in the first century BC encompass *rubor* (redness), *tumor* (swelling), *calor* (heat), and *dolor* (pain) (Majno, 1991), which arise from responses of tissue resident and blood-borne cells that are regulated by chemical signals, such as prostaglandins (PG), thromboxane (TX), leukotrienes (LT), cytokines, and chemokines. Increase in permeability of microvessels determines plasma fluid leakage and accumulation in tissues, leading to edema (Figure 1). This is followed by polymorphonuclear neutrophil (PMN) recruitment, adhesion to vascular endothelial cells, diapedesis (or transmigration), and accumulation or swarming. Their primary function is to eliminate bacteria or other damaging substances mainly *via* phagocytosis, i.e., the engulfment of foreign bodies inside intracellular vacuoles (phagosomes) and their disposition (Gordon, 2016). PMNs also release their granule contents, DNA, and chromatin proteins to form extracellular fibers that immobilize and kill bacteria. Neutrophil extracellular traps (NETs) represent a potent, innate mechanism by which PMNs prevent microbe spreading following infection (Brinkmann et al., 2004). PMNs undergo rapid apoptosis and are actively removed by macrophages (MФs) differentiated from monocytes entering as a second wave in inflamed tissues in a process termed “efferocytosis” (deCathelineau and Henson, 2003). Studies also indicate that PMNs can exit the inflammatory loci *via* lymphatic vessels or lining adipose tissue (lipopassage) (Schwab et al., 2007). MФs are also capable of clearing bacteria, pathogenic substances, and debris. Ideally, their action occurs in a non-phlogistic way and allows the resolution of inflammation (Serhan et al., 2007). Cardinal signs of resolution are: (1) limitation/cessation of PMN infiltration, (2) sequestration and counter-regulation of pro-inflammatory chemical mediators, (3) apoptosis of PMN and removal (e.g., by efferocytosis), (4) clearance of pathogens, inflammatory stimuli, and cell debris, and (5) tissue repair.

MФs are master cells in resolution since they have specialized functions, also mirrored by specific cellular and molecular phenotypes (Stables et al., 2011), that permit the clearance of pathogens, infiltrated leukocytes, or inflammatory debris and regulate the tissue remodeling or repair (Gordon, 2007). It is now evident that failure to resolve inflammation in a proper and timely manner preludes to pathology since the persistence of the phlogistic process can lead to tissue damage or systemic disease (Nathan and Ding, 2010). Therefore, what makes inflammation an “unwanted” process it is not how often or how extensive it starts, but how quickly and effectively it resolves.

## Non-Resolving Airway Inflammation in Cystic Fibrosis

Unresolved acute inflammation and chronic infection, mainly due to *P. aeruginosa*, are key mechanisms responsible for progressive airways destruction in cystic fibrosis (CF), the most common hereditary human disease (Davis et al., 1996; Cantin et al., 2015). In CF patients, airway inflammation starts early in life, persists even in the absence of detectable microbial colonization, is exaggerated in magnitude compared to the burden of infection, and does not resolve (Table 1). Despite ground-breaking advances in CF therapies obtained with the discovery of drugs that correct or potentiate the defective CF transmembrane conductance regulator (CFTR) protein, the unrelenting inflammatory response in the airways and persistent, recurrent infections remain the principal cause of progressive lung disease in patients, contributing to the high morbidity and early mortality of CF, with ~800 life losses/year (Cystic Fibrosis Foundation, 2016; Zolin et al., 2017). Remarkably, current anti-inflammatories, like ibuprofen, that block the activation phase of inflammation by inhibiting cyclooxygenase (COX)-derived prostanoids have provided little clinical benefits to patients (Lands Larry and Stanojevic, 2013), suggesting that multiple factors contribute to airway inflammation in CF. Hence, different strategies must be explored, considering that the risk/benefit ratio for anti-inflammatories in CF is particularly narrow since inflammation is required for restraining bacterial spread.

**Table 1:** Hallmarks of airway inflammation in patients with CF.

Hallmarks of airway inflammation in CF
1. Begins early in life
2. Is disproportionate to the degree of infection
3. Starts and/or persists even in the absence of infection
4. Never resolves

**Figure 2:**
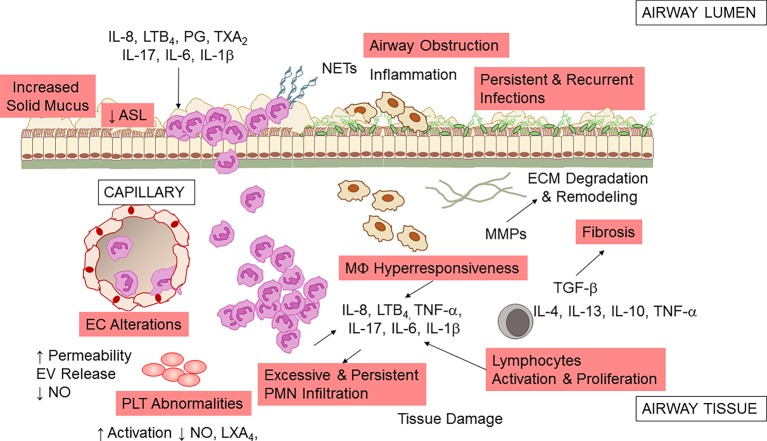
Airway inflammation in CF. A schematic view of the inflammatory response and a few of the secondary pathologic findings in CF airways. Microbial infection (by *P. aeruginosa, S. aureus, Non-tuberculosis Mycobacterium,* and other pathogens) incites a vigorous, disproportionate inflammatory response. PMNs infiltrate the airway and release proteases and oxidants that damage the airway and chemoattractants that stimulate further neutrophil influx. Although neutrophils and epithelial cells have been most intensely investigated, alterations in endothelial cells (EC), macrophages (MΦ), platelets (PLT), and T lymphocytes also play a pathophysiological role in CF airway non-resolving inflammation. See within text for further details. Not all mediators and cell types implicated in CF are shown.

The unrelenting, non-resolving lung inflammation in CF patients is participated by a number of cells, stimuli, and cellular pathways (Figure 2). Although lung of neonates with CF is structurally normal, bronchiolar mucus plugging, inflammation, and hypertrophy of submucosal gland ducts are evident as early as few months of age even without detectable infections (Bedrossian et al., 1976; Stoltz et al., 2015). Once patients with CF are challenged by bacterial or viral infection, airway inflammation is disproportionate to the degree of infection, with a high PMN infiltration and large release of pro-inflammatory molecules, such as tumor necrosis factor (TNF)-α, interleukin (IL)-8, 6, 1β (Balough et al., 1995; Bonfield et al., 1995; Noah et al., 1997; Muhlebach et al., 1999; Tirouvanziam et al., 2000; Muhlebach and Noah, 2002), prostaglandins, and LTB_4_ (Konstan et al., 1993). More importantly, lung inflammation in CF appears incapable of removing pathogens effectively and never resolves (Roesch et al., 2018).

PMNs are the predominant leukocytes contributing to airway inflammation in CF. Once recruited at CF airway tissues, they play an important defense function by phagocytosing microorganisms, secreting antimicrobial peptides, and entrapping microbes in neutrophil extracellular traps (NETs), DNA meshes formed of released DNA and proteins. However, in CF, excessive and uncontrolled neutrophil infiltration and activity have pathologic consequences, with the release of proteolytic enzymes that can degrade extracellular membrane and tissues, oxidant species that can cause structural damages to the airways and further aliment inflammation, and NETs that increase mucus viscosity (Nichols and Chmiel, 2015).

Macrophages also contribute to the non-resolving inflammation in CF airways (Bruscia and Bonfield, 2016). Data indicate that MΦs of CF patients have a hyper-responsive phenotype, producing a high amount of inflammatory cytokines when exposed to bacterial stimuli (Bruscia et al., 2009, 2011). This abnormal response may arise to basic defects in CFTR or to the persistence in bacterial colonization. CF MΦs also seem to have a defective ability to clear bacteria (possibly secondary to impaired acidification of phagosome due to CFTR dysfunction) (Di et al., 2006; Deriy et al., 2009; Del Porto et al., 2011), although this is not completely understood (Haggie and Verkman, 2007).

In addition to the well-known role in regulating vascular tone, blood fluidity, and hemostasis, platelets (PLTs) have important roles in innate immunity and inflammation. PLT can release nitric oxide (NO) and prostanoids that control leukocyte interactions with endothelial cells (EC), respond to pathogens, and interact with blood leukocytes dictating their fate and functions (Rondina et al., 2013). Clinical-based and *ex vivo* evidence of increased PLT activation in CF has been documented, suggesting that these cells can contribute to inflammation in these patients (Ciabattoni et al., 2000; O’Sullivan et al., 2005; Lindberg et al., 2018) Moreover, PLTs are important effector of resolution, since they carry enzymes and substrates required for the biosynthesis of pro-resolving mediators. PLT interactions with PMN, a key process occurring during inflammation, lead to the formation of lipoxins (LX) A_4_ (Romano and Serhan, 1992; Romano et al., 1993), the first identified specialized pro-resolving lipid mediator (SPM) that stops inflammation and promotes return to homeostasis (Romano et al., 2015). A study from Mattoscio and coworkers demonstrated that defective CFTR function expressed at PLT surfaces decreases LXA_4_ formation by inhibiting PLT LX synthase activity (Mattoscio et al., 2010). In the same study, the authors also provided compelling evidence that PLTs isolated from volunteers with CF produce significantly less NO and have an increase pro-survival activity in PMN, thus prolonging the duration of inflammation (Mattoscio et al., 2010). Critical roles of PLT in inflammation and immunity have been recently confirmed in a preclinical model of *P. aeruginosa* lung infection (Amison et al., 2018), further underlining the involvement of these cells in CF lung inflammation.

Epithelial cells form the lining of airway tissues and are the first encounter of microbes and other toxins that activate the acute inflammatory reactions. CFTR is abundantly present on the apical side of airway epithelial cells (AECs), and the absence of a functional protein causes incomplete cAMP-dependent Cl^−^ and HCO_3_^−^ secretion in airway. Therefore, mucus becomes dehydrated (Perez et al., 2006), mucins (main protein components of secretions) are tethered at apical AEC surfaces (Kreda et al., 2012; Mall, 2016; Livraghi-Butrico et al., 2017), and extracellular pH is decreased (McShane et al., 2003), thus impairing host antimicrobial defenses (see Cantin et al., 2015; Roesch et al., 2018 for a more complete review on this topic). Increased release inflammatory cytokines and chemokines through NF-κB and intracellular stress signals (e.g., Ca^2+^) have been largely documented in AEC isolated from CF patients (Weber et al., 2001; Perez et al., 2006; Hybiske et al., 2007) and can contribute to the non-resolving feature of inflammation in CF (Roesch et al., 2018).

Vascular endothelial cells (ECs) have pivotal roles in regulating inflammation, controlling the leakage of plasma proteins as well as leukocyte infiltration by releasing soluble mediators such as NO, IL-8, PG, and TX (Gimbrone, 1995). CFTR expression in EC and the involvement of CFTR in response to hypoxia have been documented (Tousson et al., 1998; Tabeling et al., 2015). In addition, clinical signs of EC dysfunctions in individuals with CF have been documented (Tousson et al., 1998; Romano et al., 2001; Poore et al., 2013). Recently, Totani and coworkers have shown that CFTR controls homeostatic functions of EC. In particular, CFTR blockade increased EC permeability and loss of membrane integrity under flow. Also, CFTR blockade suppressed NO generation and enhanced IL-8 release, possibly contributing to the sustained PMN recruitment in CF. Remarkably, in the same study, the authors showed that a combinatorial treatment with phosphodiesterase inhibitors and β2 adrenergic receptor agonists corrected CFTR-dependent EC abnormalities providing novel cellular targets for treating inflammation in CF (Totani et al., 2017).

Many dysregulated T cell subsets and related cytokines have been identified in CF lung, including Th17 that can promote PMN influx by producing IL-8 and IL-17, suggesting that T lymphocytes have important roles in lung inflammation in CF (Kushwah et al., 2013).

The introduction of CFTR correctors and potentiators allowed gaining of CFTR function in individuals carrying some mutations (Nick and Nichols, 2016; Donaldson et al., 2018). However, whether a better CFTR function translates into improvements in lung inflammation and infections is unclear. In a sub-study of the GOAL trial, Rowe and colleagues found a trend downward in abundance of CF pathogens such as *Pseudomonas* and *Staphylococcus* in the airways of study participants treated with ivacaftor, but no changes in inflammatory markers, including IL-6, -8, -1β, and free elastase in sputum (Rowe et al., 2014; Heltshe et al., 2015). In a subsequent study, Hisert and colleagues found that *P. aeruginosa* abundance in subjects treated with ivacaftor declined during the first year of treatment but started to increase afterwards. Concentrations of sputum inflammatory markers were also reduced, even if still present in huge quantities at the end of the study (Hisert et al., 2017). It is conceivable that, as infection rebounds, inflammation will eventually follow.

Given the myriad of cellular, soluble, and intracellular players that can contribute to chronic inflammation in CF, an ideal anti-inflammatory drug for these patients should target many component of this excessive inflammatory response and stimulate resolution.

## Resolution of Inflammation is an Active Process

**Figure 3:**
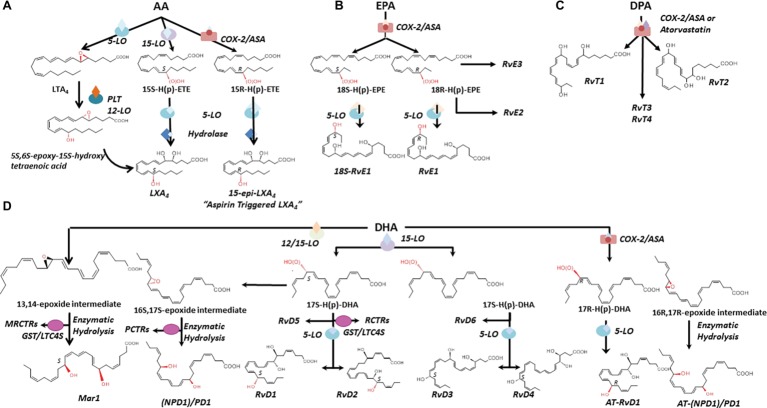
Biosynthesis and structures of SPM. The biosynthetic pathways leading to lipoxins **(A)**, E-series resolvins **(B)**, SPM derived from DPA **(C)**, D-series resolvins, protectins, and maresins **(D)** are illustrated. Structures and stereochemistries of some members of each SPM family are shown (see text for further details). AA, arachidonic acid; EPA, eicosapentaenoic acid; DPA, docosapentaenoic acid; DHA, docosahexaenoic acid; LO, lipoxygenase; COX-2, cyclooxygenase 2. MCTRs, maresin conjugate in tissue regeneration; RCTRs, resolvin conjugate in tissue regeneration; PCTRs, protectin conjugate in tissue regeneration. GST/LTC4S, glutathione-S-transferase/leukotriene C_4_ synthase.

Pioneer work by Dr. Serhan and coworkers (see Serhan and Levy, 2018 for a recent review) and from many others worldwide (Savill et al., 1989a,b; Perretti et al., 2002; Perretti and Flower, 2004; Serhan et al., 2007) has demonstrated that resolution is an *active process* regulated by specific mediators (Perretti et al., 2015) and changed the traditional view of resolution. Endogenous SPMs produced from essential polyunsaturated fatty acids (PUFAs) stop excessive PMN infiltration, counter pro-inflammatory signals, and enhance the active clearance of pathogens and death cells by MΦ. Collectively, SPMs accelerate the restitutio ad integrum; thus, they are often referred as “immunoresolvents” (Dalli et al., 2013a,b) or “agonists of resolution” (Schwab et al., 2007; Chiang et al., 2015). In addition to LX, the SPM genus includes E, D, and T series resolvins (Rv), protectins (PD), and maresins (MaR) that are biosynthesized through transcellular routes involving both resident and blood cells by lipoxygenase (LO) enzymatic activity from arachidonic acid (AA), eicosapentaenoic acid (EPA), docosapentaenoic acid (DPA), or docosahexaenoic acid (DHA) available in inflammatory exudates (Figure 3; Kasuga et al., 2008).

In human system, SPM can be found in biological specimens at pico- to nanogram levels, which are commensurate to their range of activity. SPMs are identifiable in blood (Psychogios et al., 2011; Mas et al., 2012; Colas et al., 2014; Barden et al., 2016; Dalli et al., 2017), breast milk (Weiss et al., 2013), breath condensate (Levy et al., 2007), sputum (Eickmeier et al., 2017), urine (Gangemi et al., 2003; Sasaki et al., 2015), and tears (English et al., 2017). Levels of SPM can vary in response to inflammatory stimuli (Edenius et al., 1990; Gangemi et al., 2012), vascular damage (Brezinski et al., 1992; Pillai et al., 2012), or physical exercise (Gangemi et al., 2003; Markworth et al., 2013). SPMs are also enriched in inflammatory exudates such as synovial fluids from volunteers with rheumatoid arthritis (Norling et al., 2016), skin blisters (Morris et al., 2009; Motwani et al., 2017, 2018), and bronchoalveolar lavage fluids (BALs) from patients with airway diseases (Lee et al., 1990; Karp et al., 2004; Planagumà et al., 2008; Ringholz et al., 2014). Of interest, accruing evidence indicates that SPM levels are often diminished in patients with a more severe disease, are unbalanced compared to pro-inflammatory eicosanoids, and inversely correlate with the clinical status. Several RvDs and Es, PD1, and Mar-1 are reduced in adipose tissue samples from obese subjects (Titos et al., 2016), and RvD1 is significantly lower in vulnerable areas of human atherosclerotic plaques (Fredman et al., 2016). LXA_4_ and LXA_4_ to LTB_4_ ratio are decreased in BAL from patients with CF (Karp et al., 2004; Ringholz et al., 2014). RvD1 levels are further reduced in CF patients with a worse pulmonary function (Eickmeier et al., 2017). Collectively, these studies suggest that reduced levels of SPM may contribute to the progression and worsening of human diseases.

Since resolution is an active process directed by selected mediators, the exploitation of these endogenous controllers of inflammation as therapeutics opened novel opportunities in pharmacology for the treatment of human diseases (Gilroy et al., 2004; Tang et al., 2012). For example, annexin (Anx) A1, an endogenous protein abundant in PMN granules, and melanocortins dampen inflammation and protect from tissue damage (Hecht et al., 2009; Montero-Melendez et al., 2014; Dalli et al., 2015a,b). Similarly, synthetic LX stable analogs showed anti-inflammatory and organ protective activities (Bannenberg et al., 2004; Guilford et al., 2004; Sun et al., 2009). Norling and coworkers proved the efficacy of D-series resolvins in ameliorating arthritis (Norling et al., 2016) and joint inflammation (Norling et al., 2016). AnxA1-enriched microvesicles derived from human PMN also convey anti-inflammatory, pro-resolution, and organ protective actions in experimental mandibular joint disease (Norling et al., 2011), arthritis (Headland et al., 2015), and atherosclerosis (Leoni et al., 2015). Recently, SPM proved to reduce inflammation in human volunteers undergoing UV-killed *E. coli* skin inflammation (Motwani et al., 2018) Hence, the exploration of pharmacological properties of SPM and other chemical mediators of endogenous resolution is of considerable interest (Perretti et al., 2015).

In order to define resolution in an unbiased, quantitative manner, Bannenberg et al. defined mathematical “resolution indices” by determining the cellular changes in exudates during an experimental, acute inflammatory reaction (namely local peritonitis in mice induced by injection of zymosan A particles from *S. cerevisiae*, a Toll-like receptor activator) with a fixed “time zero.” Resolution indices encompass: *T*_max_, i.e., time point of maximum PMN infiltration (Ψ_max_); *T*_50_, time necessary to achieve 50% reduction in PMN number (Ψ_50_) from Ψ_max_; resolution interval (*R*_i_ = *T*_50_ – *T*_max_), time interval between *T*_max_ and *T*_50_ (Bannenberg et al., 2005). Resolution indices have been largely used and employed in several preclinical models of inflammatory diseases for testing properties of endogenous chemical mediators or pharmacological agents (Schwab et al., 2007; Haworth et al., 2008; Navarro-Xavier et al., 2010).

## Biosynthesis of SPM

### AA Metabolome

#### Lipoxins and Aspirin-Triggered Lipoxins (ATL)

LXs (“lipoxygenase interaction products”) such as LXA_4_ and B_4_ are resulting from the enzymatic conversion of AA during cell-cell interactions (Serhan et al., 1984; Samuelsson et al., 1987). AA oxygenation by 15-LO and 5-LO, followed by enzymatic hydrolysis of hydro(peroxy)-containing intermediates, results in the formation of LXA_4_ and B_4_ (Edenius et al., 1990; Levy et al., 1993; Gronert et al., 1998) and reviewed by Recchiuti et al., 2016. In blood, AA is converted into LXA_4_ and B_4_ by the sequential activity of 5-LO (present in leukocytes) and 12-LO (abundant in platelets) (Figure 3A; Serhan and Sheppard, 1990; Romano and Serhan, 1992). In vascular cells, acetylation of COX-2 by aspirin renders this enzyme capable of converting AA into 15R-HETE, which then serves as a substrate of leukocyte 5-LO for the biosynthesis of LX containing an OH-group in the *R* configuration at C15 (Figure 3A; Claria and Serhan, 1995). These “aspirin-triggered” lipoxins (ATLs), also named 15-epi-LX, are produced in human subjects taking aspirin (Chiang et al., 2004), and mediate the anti-inflammatory actions of low-dose aspirin in healthy individuals (Morris et al., 2009). Of interest, studies from Birnbaum et al. demonstrated that atorvastatin, in addition to produce a lipid-lowering effect, promotes the generation of 15*R*-LXA_4_ *via S*-nitrosylation of COX-2 in myocardial cells (Birnbaum et al., 2006), whereas Gutierrez et al. showed that pioglitazone, an insulin-sensitizing agent, raises plasma levels of 15-epi-LXA_4_ (Gutierrez et al., 2012). Hence, aspirin, atorvastatin, and pioglitazone can activate the resolution process.

More recently, a study by Lee and coworkers revealed a new mechanism by which 15-epi-LXA_4_ biosynthesis can be activated in the nervous system. In their study, the authors found that sphingosine kinase 1 (a key enzyme that converts sphingosine into the bioactive lipid sphingosine-1-phosphate) acetylates neuronal COX-2 skewing the production of 15-epi-LXA_4_ and other SPM, resulting in an increase in phagocytosis of Aβ-amyloid by microglial cells and improvement of Alzheimer’s disease (AD)-like pathology in mice (Lee et al., 2018). Since sphingosine kinase 1 is reduced in human AD neurons (Lee et al., 2018) and several SPMs are diminished in cerebrospinal fluid from patients with AD (Nielsen et al., 2016; Zhu et al., 2016), this study provides a new framework for targeting resolution and SPM to dampen inflammation in AD.

### EPA Metabolome

#### E-Series Resolvins

RvE1 (5*S*,12*R*,18*R*-trihydoxy-6*Z*,8*E*,10*E*,14*Z*,16*E*-EPA) (Arita et al., 2005) is produced in endothelial cells by aspirin acetylated COX-2 that converts EPA into 18R-hydro(peroxy)-eicosapentaenoic acid (HEPE), then metabolized by activated leukocytes (e.g., PMN) into RvE1 (Figure 3B). Interestingly, the 18*R*-HEPE isomer was dominant to its epimer 18*S*-HEPE in plasma from volunteers given EPA, while 18*S*-HEPE was increased by aspirin administration (Oh et al., 2011b). 18*S*-HEPE can also be converted to RvE1 and RvE2 by 5-LO and LTA_4_ hydrolase (Oh et al., 2011a, 2012), and cytochrome P450 mediates the oxygenation of EPA into RvE1 (Serhan et al., 2000; Haas-Stapleton et al., 2007). RvE2 (5*S*,18-dihydroxy-EPE) is produced *via* a reduction in 5*S*-hydroperoxy, 18-hydroxy-EPE (Tjonahen et al., 2006; Ogawa and Kobayashi, 2009; Oh et al., 2011a, 2012) in resolving exudates and in human whole blood, while 18*R-*RvE3 (17*R*,18*R*-dihydroxy-5Z,8Z,11Z,13E,15E-EPE) and epimeric 17*R*,18*S*-RvE3 are biosynthesized *via* 12/15-LO in eosinophils (Figure 3B; Isobe et al., 2012, 2013).

### DPA Metabolome

EPA is not the only ω-3 fatty acid present in mammalian cells. n-3 docosapentaenoic acid (7Z,10Z,13Z,16Z,19Z-docosapentaenoic acid; n-3 DPA) is an intermediate of EPA biosynthesis from its precursor alpha-linolenic acid (Calder, 2011). Using targeted lipid mediator metabololipidomics, Dalli and coworkers identified previously unrecognized SPM derived from DPA that are congenerous to D-series Rv, PD, and MaR. The new n-3 DPA molecules include 7,8,17-trihydroxy-9,11,13,15*E,*19*Z*-DPA (RvD1_n-3 DPA_), 7,14-dihydroxy-8,10,12,16*Z,*19*Z*-DPA (MaR1_n-3 DPA_) and related isomers MaR2 and 3_n-3 DPA_ (Dalli et al., 2013a), 10R,17S-dihydroxy-7Z,11E,13E,15Z,19Z-DPA (PD1_n-3 DPA_) (Dalli et al., 2013a; Aursnes et al., 2014), and 7S,17S-dihydroxy-8E,10Z,13Z,15E,19Z-DPA (RvD5_n-3 DPA_) (Gobbetti et al., 2017). Structures of four new bioactive molecules derived from DPA and termed 13-series resolvins (RvT) were recently reported. RvTs carry a 13-carbon position alcohol, and their biosynthesis is dependent upon nitrosylation of COX-2 by atorvastatin (Figure 3C; Dalli et al., 2015a).

#### D-Series Resolvins

Earlier lipidomic studies of resolving exudates from mice administered DHA and aspirin identified DHA-derived molecules containing an OH-group at C17 (Serhan et al., 2002). By using isolated human cells and recombinant enzymes, Serhan and coworkers identified and recapitulated the entire biosynthetic pathways of D-series resolvins. Human endothelial cells exposed to hypoxia express highly amount COX-2 that converts DHA to 13-hydroxy-DHA or 17*R*-HDHA in the presence of aspirin. Both intermediates can be metabolized by human PMN to compounds termed “aspirin triggered” D-series resolvins (Serhan et al., 2002). By contrast, in the absence of aspirin, D-series resolvins with the 17*S*-OH group are predominant (Serhan et al., 2002; Hong et al., 2003). The complete organic synthesis and the stereochemistry of 17*S*-, 17*R*-RvD1, and RvD2 have been established as follows: 7*S*,8*R*,17*S*-trihydroxy-4*Z*,9*E*,11*E*,13*Z*,15*E*,19*Z*-docosahexaenoic acid (17S-RvD1), 7*S*,8*R*,17*R*-trihydroxy-4*Z*,9*E,*11*E*,13*Z*,15*E*,19*Z*-docosahexaenoic acid (17*R*-RvD1) (Sun et al., 2007), and 7*S,* 16*R*, 17*S*-trihydroxy-4*Z*, 8*E*, 10*Z*, 12*E*, 14*E*, 19*Z*-docosahexaenoic acid (RvD2) (Spite et al., 2009).

Additional members of this family have been identified: RvD3 (4*S*,11*R*,17*S*-trihydroxy-5*Z*,7*E*,9*E*,13*Z*,15*E*,19*Z*-DHA) (Dalli et al., 2013b), RvD4 (4*S*,5*R*,17*S*-trihydroxy-6*E*,8*E*,10*E*,13*E*,15*Z*,19*Z*-DHA) (Winkler et al., 2018), RvD5 (7*S*,17*S*-dihydroxy-4*Z*,8*E*,10*Z*,13*Z*,15*E*,19*Z*-DHA), and RvD6 (4*S*,17*S*-dihydroxy-5*E*,7*Z*,10*Z*,13*Z*,15*E*,19*Z*-DHA).

#### (Neuro)Protectins

Although PD1(10*R*,17*S*-dihydroxy-docosa-4*Z*,7*Z*,11*E*,13*E*,15*Z*,19*Z*-hexaenoic acid) (Serhan et al., 2006) is the founding member of this family, several isomers that also possess lower bioactivity than PD1 have also been identified: 10*S*,17*S*-diHDHA, 4*S*,17*S-*diHDHA, 7*S*,17*S*-diHDHA, and 22-hydrox-10,17*S*-docosatriene (Serhan et al., 2002; Hong et al., 2003), and an aspirin PD1 has been reported (Marcheselli et al., 2003) (10*R*,17*R*-dihydroxy-docosa-4*Z*,7*Z*,11*E*,13*E*,15*Z*,19*Z*-hexaenoic acid) (Figure 3D).

##### Maresins

Maresins (from *macrophage mediator in resolving inflammation*) are a fourth family of DHA-derived SPM (Ishida et al., 2009). The structural elucidation, complete biochemistry assignment, and total organic synthesis of two members of this family have been reported. Mar-1 (7*R,*14*S*-dihydroxy-4*Z*,8*E*,10*E*,12*Z*,16*Z*,19*Z*-DHA) and Mar-2 (13*R*,14*S*-dihydroxy-4*Z*,7*Z*,9*E*,11*Z*,16*Z*,19*Z*-DHA) are produced in tissues by MΦ and in the vasculature during PLT-PMN crosstalk through the action of 12-LO (Serhan et al., 2009; Dalli et al., 2013c; Abdulnour et al., 2014; Deng et al., 2014). *In vivo* and *in vitro* they promote resolution by increasing efferocytosis, skewing MΦ pro-resolutive phenotypes, and inhibiting PMN infiltration. They also hold organ protective and tissue-regenerative actions (Serhan et al., 2012). Mar-1 and Mar-2 receptors have not been identified yet.

##### Cysteinyl-Conjugated SPM

Lipidometabolomic profiling of murine exudates, spleens, and human fluids (including blood and breast milk) has revealed new families of SPM covalently bond to cysteine residues, collectively named SPM “conjugated in tissue regeneration” (CTR).

DHA is converted by 12-LO into 13,14-epoxy-Maresin (an intermediate of Mar-1 and Mar-2) that can be directly conjugated at C13 to glutathione by LTC4 Synthase, yielding maresin conjugated in tissue regeneration 1 (MCTR 1;13*R*-glutathionyl, 14*S*-hydroxy-4*Z*,7*Z*,9*E*,11*E*,13*R*,14*S*,16*Z*,19*Z*-DHA), the first identified cysteinyl-SPM. Subsequent cleavage of Glu and Gly residues converts MCTR1 into MCTR2 (13*R*-cysteinylglycyl, 14*S*-hydroxy-4*Z*,7*Z*,9*E*,11*E*,13*R*,14*S*,16*Z*,19*Z*-DHA), and MCRT3 (13*R*-cysteinyl, 14*S*-hydroxy-4*Z*,7*Z*,9*E*,11*E*,13*R*,14*S*,16*Z*,19*Z*-DHA) (Rodriguez and Spur, 2015a). MCTRs accelerate tissue regeneration in planaria, reduce neutrophil infiltration during *E. coli* peritonitis, and stimulate bacterial phagocytosis by MΦ (Dalli et al., 2014, 2016a,b). They also antagonize LTD_4_ binding to recombinant cysLT receptor 1 and actions in vascular cells (i.e., induction of leakage) and intact hearts (i.e., lowering of heartbeats) (Chiang et al., 2018).

Mouse and human leukocytes convert 17H(p) DHA into distinct sets of sulfide-conjugated resolvins and protectins (Dalli et al., 2015b). Attachment of glutathione at the 7,8-epoxide intermediate of RvD generates resolvin conjugate in tissue regeneration 1 (RCTR1, 8*R*-glutathionyl-7*S*,17*S*-dihydroxy-4*Z*,9*E*,11*E*,13*Z*,15*E*,19*Z*-DHA) that is in turn cleaved into RCTR2 (8*R*-cysteinylglycinyl-7*S*,17*S*-dihydroxy-4*Z*,9*E*,11*E*,13*Z*,15*E*,19*Z*-DHA) by γ-glutamyltranspeptidase and into RCTR3 (8*R*-cysteinyl-7*S*,17*S*-dihydroxy-4*Z*,9*E*,11*E*,13*Z*,15*E*,19*Z*-DHA) *via* peptidases (Rodriguez and Spur, 2017; de la Rosa et al., 2018). RCTRs stimulate tissue repair *in vivo*, enhance MΦ phagocytosis, and reduce cytokines and PMN recruitment *in vivo* and *in vitro* (de la Rosa et al., 2018).

Binding of glutathione at C16 of 17H(p)DHA produces protectin conjugated in tissue regeneration 1 (PCTR1, 16*R*-glutathionyl-4*Z*,7*Z*,10*Z*,12*E*,14*E*,16*R*,17*S*,19*Z*-DHA) that is converted into PCRT2 (16*R*-cysteinylglycinyl-4*Z*,7*Z*,10*Z*,12*E*,14*E*,16*R*,17*S*,19*Z*-DHA) and PCRT3 (16*R*-cysteinyl-4*Z*,7*Z*,10*Z*,12*E*,14*E*,16*R*,17*S*,19*Z*-DHA) (Rodriguez and Spur, 2015b; Ramon et al., 2016). In addition to the name giving tissue regenerative actions, PCTRs possess immune-regulatory actions on innate lymphoid cells during bacterial infection *in vivo* (Dalli et al., 2017).

## SPM Receptors and Intracellular Signaling

**Figure 4:**
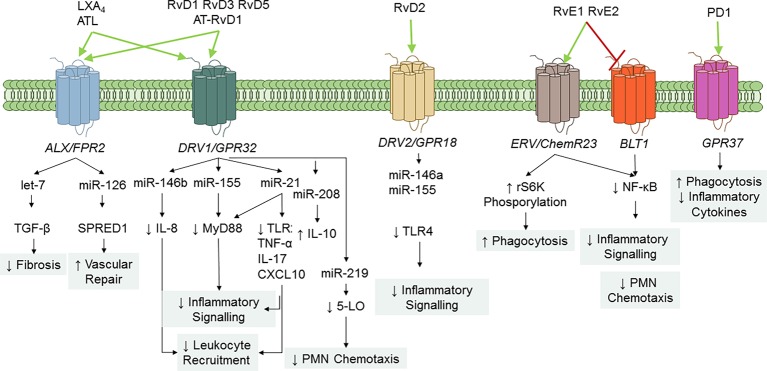
SPM GPCR and signaling pathways. To date, five specific SPM GPCRs have been identified in human and murine cells: ALX/FPR2, DRV1/GPR32, ERV1/ChemR23, DRV2/GPR18, and GPR37. Binding of SPM to their GPCR regulates cell-specific signaling pathways that ultimately diminish inflammation and enhance resolution. RvE1 also serves as a partial antagonist on leukotriene (LT) B_4_ BLT1 receptor to stop PMN infiltration.

SPMs serve principally as ligand agonists to cognate GPCRs to exert cell-specific actions that broadly regulate inflammation and resolution (Figure 4). Studies from Nigam et al. first demonstrated that LXA_4_ stimulates lipid remodeling and release of AA in PMN in a pertussis toxin-sensitive manner (Nigam et al., 1990). Fiore et al. provided the first evidence for stereoselective, specific, and reversible binding of LXA_4_ to human PMN (with a *K*_d_ ~0.5 nM), thus confirming the role of GPCR receptor(s) in conveying bioactions of LXA_4_ (Fiore et al., 1992). Subsequently, formyl peptide receptor like-1 was identified as LXA_4_-GPCR in a human neutrophil cell line (Fiore et al., 1994). This receptor has been formerly renamed ALX/FPR2 in light of its affinity for LXA_4_ (Ye et al., 2009). ALX/FPR2 is abundantly present in myeloid cells, lymphocytes, dendritic cells, and resident cells (Chiang et al., 2006). Orthologs the human ALX/FPR2 gene have been sequenced in rodents (Takano et al., 1997; Chiang et al., 2003). In addition to LXA_4_ and ATL, ALX/FPR2 is activated by AnxA1 and its N-terminal peptides bind and activate ALX/FPR2 (Perretti et al., 2002), which is the archetype of GPCR conveying both lipid and peptide pro-resolving mediators. Of interest, LXA_4_ also functions as modulator of cannabinoid receptor CB1 (Pamplona et al., 2012), which may contribute to the anti-nociceptive activities of this SPM (Svensson et al., 2007; Abdelmoaty et al., 2013). In addition, earlier studies demonstrated that 15-*epi*-LXA_4_ also binds at cysteinyl LT receptor 1 (CysLT1) with equal affinity to LTD_4_, providing further evidence for ATL in dampering CysLT signals in the vasculature (Gronert et al., 2001). Gain and loss-of-function approaches of ALX/FPR2 in human cells and in mice proved its essential role in mediating LX activities. Indeed, targeted overexpression of human ALX/FPR2 in murine myeloid cells increased the sensitivity to LXA_4_ stable analog, left-shifting the dose-response curve (Devchand et al., 2003). On the contrary, ALX/FPR2 nullified mice have an unrelenting inflammatory response, defective resolution, and lack of response to receptor ligands (Dufton et al., 2010; Norling et al., 2012; Krishnamoorthy et al., 2012). Pivotal roles of ALX/FPR2 in regulating inflammation and resolution have also been unveiled in humans. Indeed, the amounts of ATL and ALX/FPR2 expression drive magnitude and duration of the acute inflammatory response in human volunteers undergoing experimental inflammation (Morris et al., 2010). Hence, levels of ALX/FPR2 protein in tissues are fundamental to dictate the outcome of inflammation and its resolution. In this regard, Simiele et al. (2012, 2016) and Pierdomenico et al. (2015) recognized molecular basis of ALX/FPR2 transcription, identifying the core promoter sequence, transcription factors, and epigenetic mechanisms (including microRNAs) able to control ALX/FPR2 expression, and an inheritable human SNP that weakens promoter.

Arita et al. reported that RvE1 bound with high affinity (*K*_d_ = 11.3 ± 5.4 nM) and stereoselectivity to cells overexpressing the GPCR ChemR23 (formerly known as chemerin receptor) (Arita et al., 2005) and human PMN membranes (*K*_d_ ~50 nM). Interestingly, binding to PMN was displaced by homoligand RvE1 LTB_4_, and U-75302 (a selective LTB_4_ receptor 1 antagonist) (Arita et al., 2007), indicating that RvE1 binding sites on human PMN are distinct from ChemR23. Lately, direct evidence for ligand-receptor interactions of RvE1 and its epimer 18S-RvE1 was reported using ChemR23 and BLT-1 β-arrestin cells with EC50 (~6.3 pM) lower than that obtained with RvE1. In addition, the synthetic human chemerin-derived peptide (YHSFFFPGQFAFS), described as ligand for this same receptor (Wittamer et al., 2003), displaced [^3^H]-RvE1 binding, indicating that RvE1 and chemerin share binding sites on ChemR23 (Arita et al., 2005; Hasturk et al., 2006). 18*S-*RvE1 also antagonized LTB_4_-mediated BLT1 activation in BLT-1 β-arrestin assays (Oh et al., 2011b). Hence, these results indicate that RvE1 and 18*S*-RvE1 share the same site(s) of binding to human ChemR23 and BLT-1.

RvE2 exerts potent and cell-specific actions on leukocytes (Tjonahen et al., 2006; Oh et al., 2012) and binds to human PMN, ChemR23- and BLT-1 β-arrestin cells, with similar affinity to RvE1, indicating that these two SPMs partially share receptors (Oh et al., 2012).

Krishnamoorthy et al. (2010) reported the identification of RvD1 receptors in human phagocytes. In their study, RvD1 reduced actin polymerization in human PMN in a pertussis toxin sensitive manner, did not activate Ca^2+^ release, nor induced cAMP formation in human PMN. In addition, [^3^H]-RvD1 selectively bound to human PMN and monocytes with high affinity (*K*_d_ ~0.17 nM) and was displaced LXA_4_ (~60%), homoligand RvD1 (~100%), but not an AnxA1-derived peptide (Krishnamoorthy et al., 2010). Screening of a panel of GPCR established that RvD1 stopped NF-κB activation in cells overexpressing ALX/FPR2 or GPR32, but not other GPCRs (e.g., BLT1, BLT2, FPR, GPR-1, ChemR23, and CB1) treated with TNF-α. Moreover, RvD1 activated ALX/FPR2 and GPR32 in recombinant β-arrestin cells as did AT-RvD1, RvD1-carboxy-methyl ester, and a metabolically stable analog 17 (R/S)-methyl RvD1-ME (Krishnamoorthy et al., 2010). Hence, renaming GPR32 as DRV1 receptor following the International Union of Basic and Clinical Pharmacology (IUPHAR) nomenclature has been proposed (Alexander et al., 2017).

Experiments using genetically modified mice, receptor antagonists, or blocking antibodies confirmed that ALX/FPR2 and GPR32 mediate the RvD1 immunoresolving actions (Krishnamoorthy et al., 2010; Gavins et al., 2011; Hellmann et al., 2011; Recchiuti et al., 2011; Tang et al., 2012; Barnig et al., 2013; Buchanan et al., 2013; Lee and Surh, 2013; Norling et al., 2016), that encompass modification of transcription factors, microRNAs, and genes (Recchiuti et al., 2014). Human GPR32 is mostly expressed not only on PMN, monocytes, and Mɸ, but also on vascular endothelial cells (Krishnamoorthy et al., 2010). The murine ortholog of GPR32 is still unidentified, whereas it has been recently described in chimpanzees. Molecular circuits regulating GPR32 expression are unknown, while those of ALX/FPR2 have been reported and described (Simiele et al., 2012; Pierdomenico et al., 2015; Simiele et al., 2016).

Specific receptors for RvD3 and RvD4 have not yet been recognized. However, RvD3 and AT-RvD3 both stimulate GPR32, which contributes to their pro-resolving activities (i.e., stimulation of Mɸ phagocytosis) (Dalli et al., 2013b). Finally, Chiang et al. also demonstrated GPR32 activation by RvD5 (Chiang et al., 2012).

Earlier studies demonstrated that RvD2 biactions were inhibited by petussis toxin (Spite et al., 2009), suggesting association with GPCRs. Chiang et al. identified the GPCR GPR18 as a RvD2 receptor in human leukocytes by using β-arrestin cell screening, binding of [^3^H]-RvD2 to recombinant GPR18, and genetic manipulation of this receptor (Chiang et al., 2015, 2017). Based on these findings, GPR18 has been renamed as DRV2 following the IUPHAR guidelines.

Tritium-labeled PD1 binding was demonstrated in retinal pigment cells (RPEs) and human PMN (Kd ~ 30 pmol/mg protein). Also, cold PD1 showed almost complete displacement of radio-labeled PD1, while other related omega-3 fatty acid compounds gave minimal or no displacement (Marcheselli et al., 2010). Recently, Bang et al. (2018) reported that PD1 binds with high affinity to HEK293 cells transfected with GPR37, a GPCR also known as parkin-associated endothelin-like receptor highly abundant in the brain. PD1 elicited Ca^2+^ increase in GPR37-expressing cells and peritoneal MΦs. The authors also found that GPR37 was required for conveying PD1 actions, like enhancement of phagocytosis. Hence, GPR37 is, *bona fide*, a PD1 receptor. Of interest, peptide (TX14) derived from prosaposin (a neurotrophic and myelinotrophic protein) shares binding to GPR37 and intracellular signaling with PD1, suggesting that this receptor, similarly to ALX and ChemR23, can mediate pro-resolution actions of both lipid and peptide ligands.

SPM interactions with their SPM regulate several intracellular mechanisms involved in inflammation. RvD1, RvD2, and RvE1 decrease NF-κB activation, nuclear translocation, and cytokine production (Arita et al., 2005; Eickmeier et al., 2013; Chiang et al., 2017). RvE1 also signals the phosphorylation of the Akt-dependent ribosomal S6 kinase, which in turns stimulates MΦ phagocytosis (Ohira et al., 2010). Pioneer studies from Recchiuti et al. (2011), demonstrated the role of microRNAs in mediating SPM actions in resolution circuits. In murine exudate leukocytes from zymosan-induced peritonitis, RvD1 regulates miR-21, miR-146b, miR-208a, and miR-219 in a time- and GPCR-dependent manner. Identified target genes of these microRNAs include IL-8, IL-10, and 5-LO that have pivotal roles in acute inflammation (Recchiuti et al., 2011; Krishnamoorthy et al., 2012). In lung MΦ sorted from mouse lungs during *P. aeruginosa* chronic infection, RvD1 increased levels of miR-155 and miR-21 controls Toll-like receptor (TLR) expression and downstream proteins (e.g., MyD88), thus dampening signaling in MΦ that can fuel persistent inflammation (Codagnone et al., 2018). In MΦ infected with *E. coli,* RvD1 dampens the expression of pro-inflammatory genes such as COX-2 (Chiang et al., 2012) and, along with LXA_4_ and 17*R-*RvD1, reduces cytokine production by MΦ induced by endotoxins (Merched et al., 2008). Of interest, RvD2 also decreases the expression of TLR4, MyD88, and other accessory proteins in human monocytes, and these actions were partially due to the upregulation of miR-146a (Croasdell et al., 2016). Therefore, regulations of MΦ responses *via* microRNAs, NF-κB, and TLR are a common mechanism of action of SPM.

SPMs are organ protective in experimental inflammation. LXA_4_ reduces kidney fibrosis and attenuates production of proteins stimulating fibrosis (e.g., fibronectin, N-cadherin, and thrombospondin) by upregulating microRNA let-7c and suppressing transforming growth factor (TGF)-β (Brennan et al., 2013). In human EC, LXA_4_ increases the release of extracellular vesicles enriched of miR-126-5p, which, upon uptake by neighbor cells, diminishes sprouty-related EVH1 domain containing one protein and enhances wound healing (Codagnone et al., 2017). More recently, Mattè et al. found that oral administration of 17*R-*RvD1 in humanized sickle cell mice exposed to hypoxia/reoxygenation stress gave a marked increase in miR-126 and let-7 in lungs and kidneys, resulting in protection from inflammation-driven organ damage. Of interest, in the same model, 17*R-*RvD1 reduces activation of Nrf-2, a pivotal intracellular protein involved in chronic inflammation, and stimulates clearance of apoptotic PMN and damaged erythrocytes by MΦ (Matte et al., 2019). Hence, SPMs act on multiple cellular pathways to stop further inflammation, protect from tissue damage, and stimulate pro-resolutive functions of MΦ.

## Roles and Actions of SPM in CF

Several studies demonstrate that the ability of the CF lung to resolve inflammation is defective, thus contributing to the pathophysiology and progression of lung damage in patients. A study from Karp and colleagues found lower amounts of LXA_4_ in BAL of CF children as compared to non-CF pediatric patients with other respiratory infections (Karp et al., 2004), and these findings were further corroborated by Ringholz et al. (2014). Along these lines, Mattoscio and coworkers demonstrated that CFTR defects dampen LXA_4_ production during PLT:PMN interaction (Mattoscio et al., 2010). Based on these observations, acebilustat (CTX-4430), an oral inhibitor of LTA_4_ hydrolase, that prevents LTB_4_ biosynthesis has been tested in phase I and II clinical trials with volunteers with CF with the hypothesis that this compound could shut down LTB_4_ and turn on LXA_4_ biosynthesis. Adult with mild-to-moderate CF symptoms treated with acebilustat had a significant reduction in sputum PMN numbers and neutrophil elastase levels (Elborn et al., 2017). A larger phase II trial has been completed with the purpose of identifying the optimal patient population, dose, duration, and endpoints for future acebilustat trials and understanding the drug’s efficacy in patients with CF (Elborn et al., 2018).

Observation of defective SPM biosynthesis and downstream pathways in patients with CF have provided the framework for testing new anti-inflammatory drugs that work by augmenting pro-resolving mediators. Lenabasum (JBT-101) is an oral agonist of cannabinoid CB2 receptor on leukocytes that trigger the biosynthesis of LXA_4_ that resolves experimental inflammation in mice (Zurier et al., 2009). A phase IIa clinical trial of lenabasum recently completed in CF (Chmiel et al., 2017). At the end of the study, volunteers in the lenabasum arm showed a significant reduction in IL-8 and a trend downward reduced sputum neutrophil, elastase, and IgG compared to baseline. There was also a trend toward reduced risk of pulmonary exacerbations. Interestingly, recent results from Motwani et al. demonstrate that lenabasum carries potent anti-inflammatory and pro-resolving action in humans undergoing UV-killed *E. coli* skin injection (Motwani et al., 2017). In this study, lenabasum significantly reduced PMN numbers and pro-inflammatory prostanoids in exudates, whereas it increased levels of select SPM including RvD1 and LXA_4_. A multicenter phase IIb trial with volunteers 12 year of age or older in underway (NCT03451045).

Reduced 15-LO expression in nasal epithelial cells from CF patients has also been demonstrated (Jeanson et al., 2014). The exact molecular link between CFTR dysfunction and altered LO expression and/or activity remains of interest. It has also been reported that patients with CF have an unbalance between AA and DHA levels in plasma and cells (Kuo et al., 1962; Underwood et al., 1972; Gilljam et al., 1986; Freedman et al., 2004), which has prompted several studies aimed at restoring DHA levels with dietary supplementation of this omega-3 to patients (Elliott, 1976). Recently, works from Pierdomenico et al. (2017) demonstrate increased levels of the miR-181b in MΦ and epithelial cells from airways of CF individuals. Increased miR-181b leads to a reduction in ALX/FPR2 expression and blunts the ability of LXA_4_ and RvD1 to enhance bacterial clearance and epithelial integrity (Pierdomenico et al., 2017). In another study, Bensalem and coworkers found that AnxA1 was diminished in intestine from CFTR^−/−^ mice and nasal epithelial cells isolated from volunteers with CF (Bensalem et al., 2005). Also, inhibition of CFTR provokes an augmented inflammatory reaction in mice upon peritoneal injection of zymosan and delayed resolution related with reduced AnxA1 expression in peritoneal exudate leukocytes (Dalli et al., 2010). Thus, when resolution mechanisms are compromised, chronic inflammation will eventually ensue. Along these lines, restoration of AnxA1 levels with the recombinant protein corrected the overzealous inflammatory response seen with CFTR inhibition (Dalli et al., 2010).

Recent evidence signifies that SPMs convey protective biological activities to oppose excessive inflammation and tissue damage and to promote active return to homeostasis. During chronic *P. aeruginosa* infection in mice, RvD1 reduces PMN influx, dampens bacterial load, and ameliorates clinical sign of pathology. In addition, RvD1 also encourages *P. aeruginosa* clearance by human MΦ and PMN and *in vivo* shortened the time required to resolve inflammation. Of note, RvD1 showed comparable effects to ciprofloxacin treatment (the reference antibiotic lung exacerbation treatment in CF patients) in reducing both bacterial titer and leukocyte infiltration and demonstrated additional benefits to mono antibiotic therapy. Several cytokines and chemokines that are increased in CF airways were also diminished in mice bearing chronic *P. aeruginosa* infection by RvD1 treatment, including IL-8, IL-1β, and IL-17. RvD1 also exhibits pro-resolutive and protective actions in lung tissue since it strikingly lowers mucus metaplasia, parenchymal inflammation, and leukocyte infiltration in long-term infected *P. aeruginosa* mice (Codagnone et al., 2018). Consistently, LXA_4_ stable analog reduces neutrophil recruitment and bacterial burden in short-term *P. aeruginosa* murine models of lung infection (Karp et al., 2004). Along these lines, RvD2 reduces polymicrobial sepsis severity in mice (Spite et al., 2009), RvD1 diminishes inflammation in pneumonia triggered by viral and bacterial co-infection (Wang et al., 2017), whereas MaR1 and RvD3 enhance *E. coli* phagocytosis by MΦ (Colas et al., 2016). Therefore, confining excessive inflammation and boosting host defense against pathogens are crucial SPM bioactions during resolution.

Many of actions exerted by SPM to limit inflammation and infection were also recapitulated with isolated human cells, such as the ability to enhance phagocytosis of bacteria by leukocytes (Chiang et al., 2012; Colas et al., 2014; Pierdomenico et al., 2017; Codagnone et al., 2018). In addition, SPMs skew MΦ from a pro-inflammatory to a pro-resolutive phenotype (Dalli and Serhan, 2012; Recchiuti et al., 2014; Pistorius et al., 2018), for instance, enhancing the expression on MΦ of surface receptors involved in the uptake of apoptotic cells (Matte et al., 2019).

SPMs target epithelial cells to regulate ion transport. LXA_4_ enhances CFTR-independent Cl^−^ efflux from CF bronchial epithelial cells and inhibits Na^+^ reabsorption, thus restoring the airway surface hydration (ASL) that is important for mucociliary clearance (Verriere et al., 2012; Al-Alawi et al., 2014; Higgins et al., 2016; Ringholz et al., 2018). RvD1 also enhances ASL height in human CF bronchial cells by reducing an amiloride-sensitive Na^+^ channel (Ringholz et al., 2018). In airway epithelia exposed to bacterial infection *in vitro*, LXA_4_ and RvD1 proved to protect from cell injury, strengthen tight junction integrity, and reduce IL-8 production (Grumbach et al., 2009; Higgins et al., 2016; Ringholz et al., 2018).

SPMs counter inflammatory responses occurring in the vasculature. RvD1 reduces IL-1β-induced vascular permeability of EC and edema formation in lungs (Codagnone et al., 2018) and limits PMN adhesion on EC and diapedesis (Sun et al., 2007; Norling et al., 2012), LXA_4_ and RvD2 stimulate NO production that stops leukocyte interactions with EC (Paul-Clark et al., 2004; Spite et al., 2009). LXA_4_ and B_4_ counter LTB_4_-induced PMN migration (Papayianni et al., 1996), while RvE1 among SPM has the unique property of diminishing the number of PLT:leukocyte aggregates in human whole blood (Dona et al., 2008) and ADP-induced PLT aggregation and activation (Fredman et al., 2010), which are enhanced in patients with CF.

Emerging evidence signifies now that SPMs are safe and effective in treating inflammatory diseases in humans. A small clinical trial with infants with eczema showed that topical application of a LXA_4_ stable analog was as potent as mometasone furoate in reducing disease severity, eczema area, and clinical scores, improving the quality of life of patients (Wu et al., 2013). More recently, Kong and colleagues reported that LXA_4_ methyl ester was superior to corticosteroids in improving lung function of children with asthma and was well tolerated (Kong et al., 2017). Finally, SPM proved to reduce neutrophil infiltration and bacterial endotoxins in volunteers subjected to UV-killed *E. coli* skin inflammation (Motwani et al., 2018).

Collectively, these data indicate that SPMs act at multiple levels on cells involved in the pathophysiology of CF airway inflammation and proved effective in preclinical models and clinical trials to stimulate resolution of chronic infection and inflammation, thus opening the road for SPM-based human resolution pharmacology.

## Conclusion

Anti-inflammatory drugs remain an area of intense research in CF, since it is unclear if and to what extent CFTR modulators will have a positive effect on the incipit and persistence of airway inflammation in patients. It is unlikely that these drugs will fully reverse the functional and structural damages present in patients with established disease or carry difficult to correct mutations. The development of drugs that stop excessive inflammation and promote resolution must proceed along with the identification of more suitable biochemical or cellular markers of effectiveness in patients. Furthermore, several aspects must be considered when evaluating new anti-inflammatories for CF: (1) mechanisms of actions and target cells/pathways, which should be broad and disease-related; (2) novelty of mode(s) of action with respect to traditional drugs used to limit inflammation (e.g., ibuprofen and steroids) that have provided little clinical benefits; (3) efficacy in appropriate preclinical models, including isolated cells, organs-on-a-chip derived, and biological samples (plasma, BAL) derived from patients, as well as animal systems; (4) toxicity; (5) *in vivo* impact on host defense mechanisms that are essential for containing infections; and (6) bioavailability and delivery methods that should take into account patients with lower compliance such as children.

**Figure 5:**
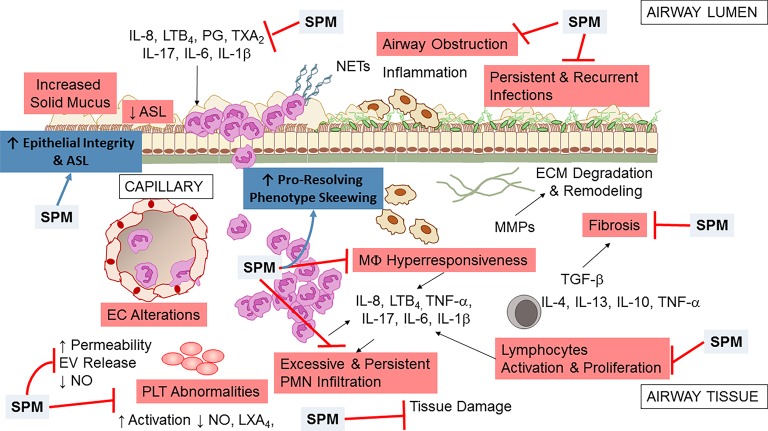
Multipronged actions of SPM in CF airway disease. Broad actions of SPM relevant to CF airway disease encompass both anti-inflammation (limitation of further PMN infiltration, reduction in cytokine production, and decrease in lymphocyte, EC, and PLT activation) and pro-resolution (enhancement of MΦ phagocytosis and bacterial clearance, promotion of tissue repair, restoration of epithelial barrier integrity). See within text and references for further details.

Data from *in vitro* and *in vivo* studies indicate that SPMs are multi-pronged, potent regulators of inflammation and resolution, acting on multiple cell and molecular targets to limit the unwanted persistence of inflammation and tissue damage and accelerating the return to homeostasis (Figure 5). In addition, emerging results from clinical trials reporting safety and efficacy of SPM and molecules that stimulate their production encourage envisaging SPM as candidate drugs for treating chronic inflammation and infection in CF patients.

## Author Contributions

AR wrote the first draft of the manuscript. All the authors have contributed to the final version of this review.

### Conflict of Interest Statement

The authors declare that the research was conducted in the absence of any commercial or financial relationships that could be construed as a potential conflict of interest.
